# Associations between mortality from COVID-19 and other causes: A state-level analysis

**DOI:** 10.1371/journal.pone.0281683

**Published:** 2023-03-06

**Authors:** Anneliese N. Luck, Andrew C. Stokes, Katherine Hempstead, Eugenio Paglino, Samuel H. Preston

**Affiliations:** 1 Department of Sociology and Population Studies Center, University of Pennsylvania, Philadelphia, PA, United States of America; 2 Department of Global Health, Boston University School of Public Health, Boston, MA, United States of America; 3 Robert Wood Johnson Foundation, Princeton, NJ, United States of America; Non-Communicable Diseases Research Center, Endocrinology and Metabolism Research Institute, Tehran University of Medical Sciences, ISLAMIC REPUBLIC OF IRAN

## Abstract

**Background:**

During the COVID-19 pandemic, the high death toll from COVID-19 was accompanied by a rise in mortality from other causes of death. The objective of this study was to identify the relationship between mortality from COVID-19 and changes in mortality from specific causes of death by exploiting spatial variation in these relationships across US states.

**Methods:**

We use cause-specific mortality data from CDC Wonder and population estimates from the US Census Bureau to examine relationships at the state level between mortality from COVID-19 and changes in mortality from other causes of death. We calculate age-standardized death rates (ASDR) for three age groups, nine underlying causes of death, and all 50 states and the District of Columbia between the first full year of the pandemic (March 2020-February 2021) and the year prior (March 2019-February 2020). We then estimate the relationship between changes in cause-specific ASDR and COVID-19 ASDR using linear regression analysis weighted by the size of the state’s population.

**Results:**

We estimate that causes of death other than COVID-19 represent 19.6% of the total mortality burden associated with COVID-19 during the first year of the COVID-19 pandemic. At ages 25+, circulatory disease accounted for 51.3% of this burden while dementia (16.4%), other respiratory diseases (12.4%), influenza/pneumonia (8.7%) and diabetes (8.6%) also contribute. In contrast, there was an inverse association across states between COVID-19 death rates and changes in death rates from cancer. We found no state-level association between COVID-19 mortality and rising mortality from external causes.

**Conclusions:**

States with unusually high death rates from COVID-19 experienced an even larger mortality burden than implied by those rates alone. Circulatory disease served as the most important route through which COVID-19 mortality affected death rates from other causes of death. Dementia and other respiratory diseases made the second and third largest contributions. In contrast, mortality from neoplasms tended to decline in states with the highest death rates from COVID-19. Such information may help to inform state-level responses aimed at easing the full mortality burden of the COVID-19 pandemic.

## Introduction

Public health crises, such as the current COVID-19 pandemic, can pose threats to health and mortality beyond those directly attributable to the disease causing the crisis itself. For example, during the 1918 flu epidemic in the United States, the country faced higher levels of mortality not only from influenza, but from other respiratory diseases and circulatory diseases [1–6]. There are many reasons to anticipate that the COVID-19 pandemic may have a similar impact on mortality from other causes of death. These include disruptions in healthcare and treatment, worsened economic conditions and financial hardship, as well as synergies between COVID-19 and other diseases. Increases in causes of death other than COVID-19 may also partially reflect misdiagnosis of COVID-19 deaths due to inconsistencies in diagnostic and death certification practices [7].

Research on the COVID-19 pandemic has primarily focused on deaths directly attributed to COVID-19 as the underlying cause of death on the death certificate [8]. Less work examines increases in mortality from causes of death other than COVID-19, despite indications that this increase made up a consequential share of the increase in all-cause mortality experienced during the pandemic. Recent estimates suggest that increases in non-COVID-19 mortality accounted for between 12% to 28% of the increase in all-cause mortality experienced during the first year of the pandemic [9–11], with heart disease, diabetes, dementia, and external causes of death implicated as the key drivers of this increase [8, 12–15].

However, most of this emerging work on increases in non-COVID-19 mortality has focused on the national level, despite the great deal of spatial variation in health and mortality across the United States [16–18]. In particular, mortality differences between states have become increasingly salient over the past several decades [19–21]. Indeed, emerging research has begun to document state-level variation in COVID-19 infection and mortality, as well as in responses to the pandemic [22, 23]. Yet the literature focused on mortality during the pandemic for causes other than COVID-19 has generally neglected the spatial patterning of the pandemic’s impact, while other studies highlight salient spatial patterns in pandemic mortality without paying attention to causes of death [9, 10].

The present paper merges these two approaches, with the objective of examining the spatial patterns in mortality change from major causes of death between March 1, 2019 and February 28, 2021. To capture a more complete picture of the association between the COVID-19 pandemic and mortality across the United States, we ask whether states with the highest burden of mortality directly attributable to COVID-19 also had higher mortality from other causes of death. We examine state-level associations between COVID-19 age-standardized death rates and changes in age-standardized death rates for selected causes of death. In doing so, this study captures spatial patterns in cause of death relationships observed during the first year of the pandemic, enabling a better understanding of the full mortality impact of the COVID-19 crisis.

## Methods

### Data sources

We obtained provisional data on all-cause and cause-specific mortality from CDC Wonder [24]. Data were queried from the provisional mortality statistics tool by five-year age group, underlying cause of death, and state of residence of the decedent for the period March 2019 through February 2021 as reported by February 6, 2022. We incorporated an approximate 12-month lag to account for delays in mortality reporting, especially for external causes of death [25]. Additionally, mid-year population estimates for 2019 and 2020 by age and state were obtained from the Census Bureau’s Vintage 2020 state-level population estimates [26].

### Cause of death categorization

We assigned deaths to COVID-19 if the International Statistical Classification of Diseases and Related Health Problems, Revision Ten, (ICD-10) code U07.1 appeared as the underlying cause of death on the death certificate [27]. Deaths from causes other than COVID-19 were classified into categories to facilitate analyses, which included circulatory disease, diabetes, Alzheimer’s disease and related dementias (ADRD), malignant neoplasms, influenza and pneumonia, other respiratory diseases, external causes, other natural causes, as well as a separate category for signs and symptoms not elsewhere classified.

Building on our previous work on cause-specific mortality during the pandemic [27], this list of selected causes was largely based on the original cause-specific mortality data published by the CDC [28]. In selecting causes of death for analysis, we chose to focus on broad categories rather than individual causes of death to reduce risk of data suppression in states with low cause-specific death counts. These causes of death, excluding the catch-all other natural cause category, account for approximately 85% of all-cause mortality in both periods (**Table 1**). Many of the included causes overlap with the CDC’s list of leading causes of death in the United States [29] or appear on the CDC’s recently published list of COVID-19 co-morbid conditions [30], including influenza and pneumonia, other respiratory diseases, circulatory diseases, malignant neoplasms, dementia, and diabetes. In addition, we include external causes and signs and symptoms not classified to build off previous work that has examined these causes of death in their analyses [8, 12, 32, 56]. We also offer a supplementary analysis investigated a more detailed list of select external causes commonly examined in the literature, including drug overdose, homicide, suicide, and transport accidents. A full table of ICD-10 codes for each cause of death category can be found in the **S1 Table in S1 Appendix**.

**Table 1:** National distribution of all-cause mortality by select causes of death, ages 25+.

	3/2019-2/2020		3/2020-2/2021	
	ASDR	% of all-cause	ASDR	% of all-cause
All-Cause	619.1	100.0%	749.7	100.0%
COVID-19	0.0	0.0%	105.7	14.1%
Non-COVID-19	619.1	100.0%	643.9	85.9%
Dementia	60.0	9.7%	66.3	8.8%
Diabetes	19.2	3.1%	22.5	3.0%
Circulatory Disease	192.9	31.2%	201.8	26.9%
Influenza & Pneumonia	10.9	1.8%	10.2	1.4%
Other Respiratory	48.8	7.9%	45.1	6.0%
Malignant Neoplasms	132.3	21.4%	128.9	17.2%
Signs Not Classified	6.1	1.0%	6.5	0.9%
External Causes	49.1	7.9%	55.0	7.3%
Other	98.8	16.0%	106.7	14.2%

Note: ASDR = age-standardized death rates.

Cause-specific mortality data is suppressed where the death count for the period was less than 10. However, our use of US states as the unit of analysis and focus primarily on leading causes of deaths resulted in marginal suppression in our data. Across the set of nine inclusive causes of death included in the study, less than 1.4% of all-cause deaths were unaccounted for, driven primarily by suppression at younger age groups and in less populous states. Given the limited suppression in our data, we assume no deaths occurred when data was suppressed for a given age group, state of residence, and cause of death combination. We instead address suppression through the use of weighted regressions, which reduces the influence of less populous states where data suppression was most likely.

### Age-standardized death rates

The study compared age-standardized death rates for age groups 25+, 65+, and 25–64 observed in the first full year of the pandemic (March 2020 to February 2021) to baseline pre-pandemic rates during the prior corresponding period (March 2019 to February 2020). Although our time period spanned February to March, we used July 1 population estimates as the mid-period estimate of the population given the unavailability of monthly-level data. Age-specific death rates were then age-standardized using the mid-year 2020 national age distribution for population aged 25+ as well as for ages 25–64 and 65+ [31].

### Analytic approach

Our units of observation are US states. States are a convenient vehicle for investigating disease interrelations since many programs and policies addressed to harness the pandemic were implemented at the state level. Further, states are generally large enough that data suppression due to small numbers in NCHS data releases is relatively uncommon, in contrast to county-level data. However, data suppression may appear in relation to mortality from less prevalent causes of death in our study, such as the more detailed categories of external causes.

All death rates in this analysis were age-standardized using the age distribution of the United States in 2020 in 5-year age groups. The principal focus was on age-standardized death rates (per 100,000) for ages 25+. We also examined age-standardized rates at ages 65+, where chronic diseases dominate, and at working ages 25–64, where external causes of death are more common.

We examine relations between changes in mortality for a particular cause of death and COVID-19 mortality using weighted linear regression analysis. Each state’s observation is weighted by the corresponding size of the state’s population in the appropriate age span. Weighted regression is used so that results better reflect the population distribution of the United States.

The basic regression equation is:

$$\Delta M_{ik}=\alpha_{i}+\beta_{i}\cdot M_{ck}+\varepsilon ,$$

where

Δ*M*_*ik*_ = change in age-standardized death rate from cause i in state k

*M*_*ck*_ = age-standardized death rate from COVID-19 in state k

*α*_*i*_ = constant term expressing change in mortality from cause i that is unrelated to COVID-19 mortality

*β*_*i*_ = increase in mortality from cause i per unit increase in mortality from COVID-19

We focus on absolute changes in age-standardized death rates as the most direct measure of the change in frequency of death per person. In doing so, the sum of *β*_*i*_’s across a set of mutually exclusive and exhaustive causes of death, excluding COVID-19, will add to the *β*_*i*_ coefficient when all non-COVID-19 mortality is regressed on COVID-19 mortality, *M*_*ck*_. In this fashion, the relation between mortality from non-COVID-19 causes and COVID-19 mortality can be uniquely decomposed into relations for various causes of death. The contribution of a particular cause of death to the relation between COVID-19 and all-cause mortality is assessed by the ratio of the cause-specific *β*_*i*_ to the all-cause *β*_*i*_. Additional regressions were also implemented in which age-standardized death rates were standardized (mean = 0 and standard deviation = 1) to further investigate the magnitude of relationship between increases in cause-specific and COVID-19 mortality. All models were implemented using the *stats* package in R.

## Results

### Cause-specific mortality by state

**Fig 1:**
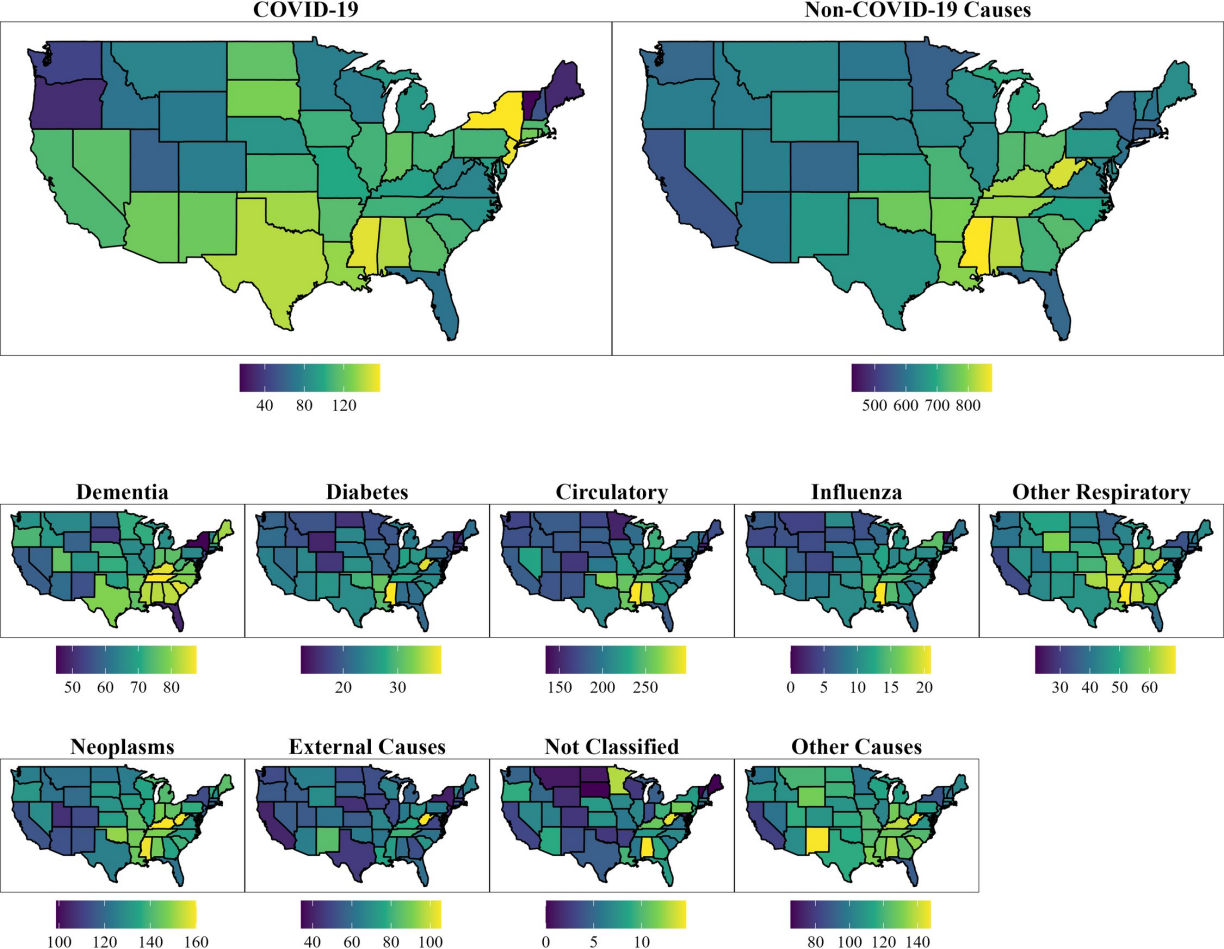
State distribution of cause-specific ASDR for ages 25+ from 3/2020-2/2021. Note: ASDR = age-standardized death rates. Units are deaths per 100,000.

**Table 2** presents the estimated all-cause, COVID-19, and non-COVID-19 age-standardized death rates (ASDR) during the year prior to the pandemic (March 1, 2019 to February 28, 2020) and during the first full year of the pandemic (March 1, 2020 to February 28, 2021) across all 50 US states and the District of Columbia. **Fig 1** displays the state distribution of age-standardized death rates for COVID-19 and non-COVID-19, as well as across all nine selected causes of death across US states in the first year of the pandemic.

**Table 2:** All-cause, COVID-19, and non-COVID-19 ASDR for ages 25+ by state and time period.

		All-Cause		COVID-19		Non-COVID-19	
State	Abbreviation	3/2019-2/2020	3/2020-2/2021	3/2019-2/2020	3/2020-2/2021	3/2019-2/2020	3/2020-2/2021
Alabama	AL	768.6	964.5	-	139.7	768.6	824.8
Alaska	AK	588.2	641.9	-	34.3	588.2	603.9
Arizona	AZ	564.1	732.1	-	123.8	564.1	608.3
Arkansas	AR	750.9	895.0	-	113.8	750.9	781.1
California	CA	521.4	659.3	-	110.9	521.4	548.4
Colorado	CO	561.3	656.1	-	74.8	561.3	581.3
Connecticut	CT	559.8	683.7	-	124.5	559.8	559.1
Delaware	DE	621.0	747.2	-	91.0	621.0	654.6
District of Columbia	DC	610.5	784.9	-	129.1	610.5	654.3
Florida	FL	558.0	648.2	-	68.8	558.0	579.4
Georgia	GA	673.8	834.2	-	115.1	673.8	719.2
Hawaii	HI	434.6	442.1	-	14.7	434.6	426.5
Idaho	ID	616.9	688.1	-	69.3	616.9	618.1
Illinois	IL	614.0	751.6	-	107.3	614.0	644.3
Indiana	IN	720.1	854.4	-	119.3	720.1	735.1
Iowa	IA	644.2	746.0	-	105.5	644.2	640.1
Kansas	KS	667.8	779.8	-	106.7	667.7	673.1
Kentucky	KY	791.4	910.8	-	95.5	791.4	815.3
Louisiana	LA	734.4	928.0	-	133.5	734.4	794.5
Maine	ME	659.6	680.0	-	29.7	659.6	648.7
Maryland	MD	605.2	728.0	-	95.0	605.2	632.9
Massachusetts	MA	571.6	675.8	-	112.1	571.6	563.7
Michigan	MI	674.4	794.2	-	91.7	674.4	702.5
Minnesota	MN	568.6	643.6	-	75.4	568.6	568.1
Mississippi	MS	809.7	1025.9	-	150.0	809.7	875.8
Missouri	MO	690.6	824.0	-	100.0	690.6	724.0
Montana	MT	628.6	718.0	-	78.7	628.6	638.4
Nebraska	NE	617.5	717.0	-	88.6	617.5	627.5
Nevada	NV	639.6	771.1	-	115.8	639.6	655.3
New Hampshire	NH	601.8	655.4	-	52.7	601.8	601.6
New Jersey	NJ	569.8	750.9	-	152.9	569.8	598.0
New Mexico	NM	636.7	790.8	-	122.2	636.7	668.5
New York	NY	533.8	721.2	-	156.7	533.8	564.6
North Carolina	NC	662.8	773.3	-	86.8	662.8	686.4
North Dakota	ND	621.5	721.2	-	117.6	621.5	602.9
Ohio	OH	714.9	849.9	-	107.5	714.9	742.4
Oklahoma	OK	747.3	905.0	-	136.1	747.3	768.9
Oregon	OR	608.3	650.1	-	31.8	608.3	618.1
Pennsylvania	PA	648.6	774.8	-	107.9	648.6	666.9
Rhode Island	RI	612.9	724.5	-	120.2	612.9	603.2
South Carolina	SC	692.9	847.6	-	108.7	692.9	738.9
South Dakota	SD	630.7	757.6	-	127.2	630.7	628.4
Tennessee	TN	763.7	910.4	-	106.5	763.7	803.9
Texas	TX	629.0	803.7	-	140.3	629.0	663.4
Utah	UT	601.0	679.5	-	60.0	601.0	619.5
Vermont	VT	596.7	647.5	-	16.3	596.7	628.6
Virginia	VA	615.7	715.7	-	79.2	615.7	636.5
Washington	WA	579.1	622.1	-	42.9	579.0	579.2
West Virginia	WV	808.4	929.0	-	80.9	808.4	847.9
Wisconsin	WI	634.0	725.6	-	75.2	634.0	650.3
Wyoming	WY	631.9	735.7	-	72.9	631.9	660.6

Note: ASDR = age-standardized death rates. 2019 year of the pandemic refers to March 2020 to February 2021. Line reflects weighted linear regression where weights are the corresponding size of population in state i. *β* represents the unit increase in non-COVID-19 ASDR associated with a 1 per 100,000 unit increase in COVID-19 ASDR. States and their associated abbreviations can be found in Table 1.

As seen in **Table 2**, the five states with the highest levels of all-cause mortality pre-pandemic were located in the South. This geographic patterning remained consistent during the first year of the pandemic, with these Southern states consistently facing the highest levels of both all-cause and non-COVID-19 mortality [**Fig 1**]. However, more geographic variation emerged among states with the highest levels of COVID-19 mortality. Though most of the highest COVID-19 mortality states were located in the South, New York and New Jersey topped the list as leaders in COVID-19 mortality. These two states faced dramatic shifts in mortality from 2019 to 2020, having the highest death rates from COVID-19 in the country during the first year of the pandemic yet the being in among the ten states with the lowest mortality in the pre-pandemic year.

The levels of COVID-19 mortality during the first year of the pandemic also proved much more variable across states than non-COVID-19 or all-cause mortality [**Table 1**]. Across both years, the state with the highest levels of all-cause and non-COVID-19 mortality (Mississippi) had death rates 1.9–2.3 times higher than the state with the lowest all-cause mortality rate (Hawaii). From March 2020 to February 2021, however, the states with the highest levels of COVID-19 mortality (New York and New Jersey) had death rates from COVID-19 ranging between 9.4 to 10.7 times higher than those with the lowest levels of COVID-19 mortality (Hawaii and Vermont).

**Fig 1** shows that similar geographic patterns generally hold across the select causes of death examined in this study. In 2020, across nearly every cause of death, the five states with the highest levels of mortality are located in the Southern region of the United States, with the only exceptions being influenza and pneumonia, external cause deaths, other signs and symptoms, and all other causes.

### COVID-19 and changes in cause-specific mortality

**Fig 2:**
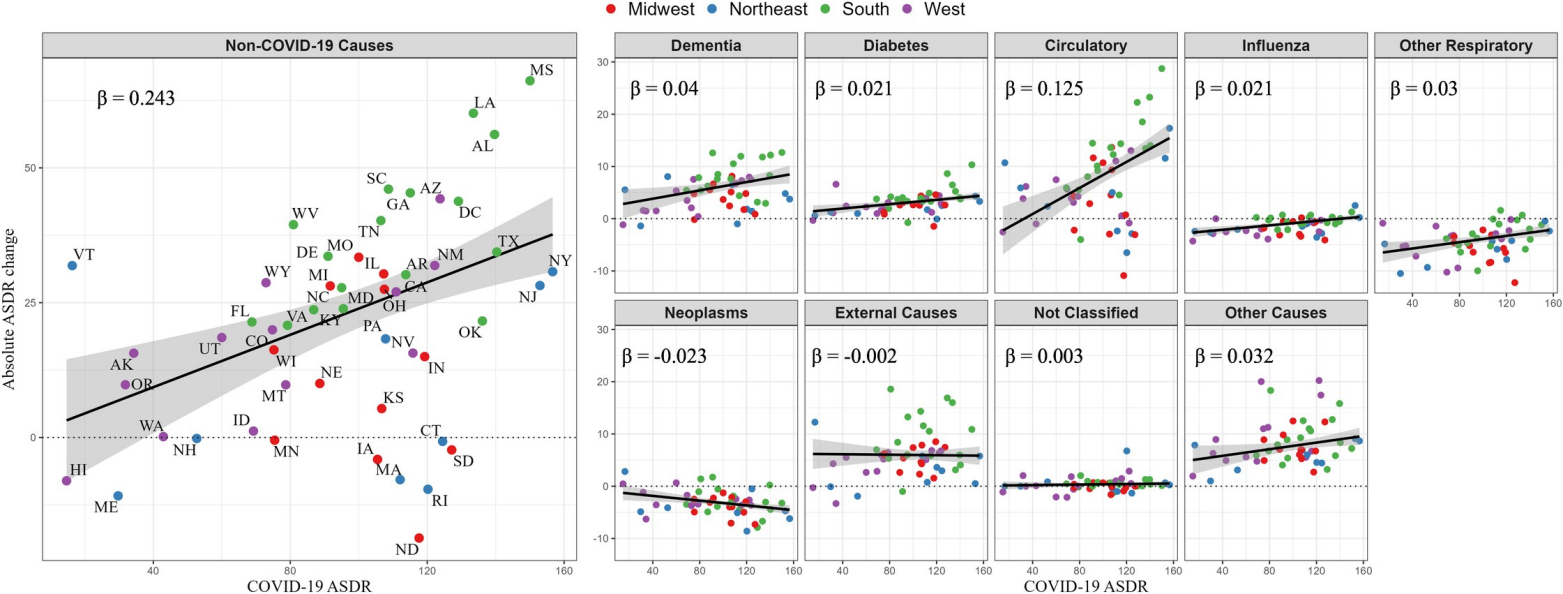
Association between state-level COVID-19 mortality and change in cause-specific mortality, ages 25+. Note: ASDR = age-standardized death rates. Change is measured as the age-standardized death rate in first full year of the pandemic (March 2020 to February 2021) relative to the same period a year prior (March 2019 to February 2020). Line reflects weighted linear regression where weights are the corresponding size of population in state i. Points above the dotted line indicates states with increases in cause-specific age ASDR, while below indicates decreases. *β* represents the change in ASDR for a particular cause of death associated with a 1 per 100,000 unit increase in COVID-19 ASDR. States are identified in the non-COVID-19 panel, with the associated abbreviations found in Table 1.

Next, we directly examine the relationships between age-standardized death rates from COVID-19 during the first full year of the pandemic (March 1, 2020 to February 28, 2021) and changes in age-standardized death rates from other causes of death between this period and the prior year (March 1, 2019 to February 28, 2020). The relationships are plotted in **Fig 2**. The line in the Figure was fitted by weighted linear regression, the parameters of which are shown in **Table 3**.

**Table 3:** Regression of state-level change in cause-specific mortality on COVID-19 mortality.

	Ages 25+					Working Ages (25–64)					Older Ages (65+)				
				β Decomposition					β Decomposition					β Decomposition	
	α	β	p-value	All-	Non-	α	β	p-value	All-	Non-	α	β	p-value	All-	Non-
				Cause	COVID-19				Cause	COVID-19				Cause	COVID-19
All-Cause	0.02	1.240	<0.001	~100%	-	14.35	1.180	<0.001	~100%	-	-25.07	1.199	<0.001	~100%	-
COVID-19	0.00	1.000	<0.001	80.7%	-	0.00	1.000	<0.001	84.7%	-	0.00	1.000	<0.001	83.4%	-
Non-COVID-19	-0.39	0.243	<0.001	19.6%	~100%	13.99	0.190	0.022	16.1%	~100%	-25.33	0.199	0.002	16.6%	~100%
Dementia	2.23	0.040	0.008	3.2%	16.4%	-0.04	0.004	0.134	0.3%	2.0%	11.58	0.042	0.035	3.5%	21.1%
Diabetes	1.12	0.021	<0.001	1.7%	8.6%	0.81	0.030	0.001	2.5%	15.5%	3.62	0.014	0.017	1.2%	7.2%
Circulatory Disease	-4.09	0.125	<0.001	10.1%	51.3%	3.29	0.072	0.009	6.1%	37.9%	-15.85	0.106	<0.001	8.8%	52.9%
Influenza & Pneumonia	-2.96	0.021	<0.001	1.7%	8.7%	-0.52	0.015	0.002	1.3%	8.1%	-9.50	0.019	0.001	1.6%	9.7%
Other Respiratory	-6.91	0.030	0.005	2.4%	12.4%	-0.53	0.021	0.016	1.8%	11.2%	-23.48	0.023	0.063	1.9%	11.6%
Malignant Neoplasms	-0.94	-0.023	0.003	-1.8%	-9.4%	-1.40	0.009	0.454	0.7%	4.6%	2.68	-0.037	<0.001	-3.1%	-18.4%
Signs Not Classified	0.12	0.003	0.520	0.2%	1.0%	0.06	0.010	0.100	0.8%	5.3%	-0.04	0.002	0.680	0.1%	0.8%
External Causes	6.21	-0.002	0.879	-0.2%	-0.9%	7.49	0.006	0.909	0.5%	2.9%	0.22	0.002	0.748	0.1%	0.8%
Other	4.55	0.032	0.029	2.6%	13.0%	4.74	0.027	0.262	2.3%	14.4%	4.77	0.030	0.028	2.5%	15.2%

Note: Table presents OLS regression of state-level absolute change in cause-specific age-standardized death rates (per 100,000) in the first full year of the pandemic (March 2020 to February 2021) relative to the same period a year prior (March 2019 to February 2020) on the COVID-19 age-standardized death rate in the first year of the pandemic. Regressions were weighted by the state population in each age group. Coefficients for all-cause and non-COVID-19 ASDR change are decomposed into the contribution of the select causes of death. Inconsistencies across age groups are a result of the presence of inter-group effects of COVID-19 mortality (i.e. COVID-19 mortality of age group 25–64 affecting mortality of age group 65+ and vice versa), see replication file for additional details.

The coefficient of the regression of the change in all-cause mortality on COVID-19 mortality is 1.24, meaning that for each one-unit increase in COVID-19 mortality, all-cause mortality rose by 1.24 units [**Table 3**]. This result implies that each increase of one unit in COVID-19 mortality was accompanied by an increase of 0.24 units in mortality from all other causes of death combined, so that other causes represent 0.24/1.24 or 19.6% of the total impact of COVID-19 on all-cause mortality.

The causes of death that were associated with an increase in COVID-19 mortality can be gauged by the magnitude of the beta coefficients in cause-specific regressions, which represent the change in mortality for a particular cause of death per unit change in COVID-19 mortality. Across causes of death, these coefficients sum to the beta of 1.24 pertaining to all-cause mortality. The marginal difference between the direct regression of all-cause mortality (1.243) and sum of betas across causes of death (1.240) reflect rounding error and/or suppression of data with small sample sizes. The highest coefficient, 0.125, pertained to circulatory disease. This result suggests that for every 1 unit (1 per 100,000) increase in COVID-19 mortality, there was an associated increase of 0.125 per 100,000 in circulatory mortality. This coefficient was followed by that of dementia (0.040), other respiratory diseases (0.030) and diabetes (0.021). These four causes account for nearly 89% of the 0.243 increase in non-COVID-19 causes of death per unit increase in COVID-19 mortality. **Table 3** shows that the relationship between mortality from dementia and from COVID-19 is essentially limited to ages 65+. As a proportion of the increase of 19.6% in all-cause mortality attributable to non-Covid causes of death ages 25+, circulatory disease accounts for 52.9% of this burden while dementia (21.1%), other respiratory diseases (11.6%), influenza/pneumonia (9.7%) and diabetes (7.1%) also contribute.

Malignant neoplasms are the only cause of death showing a significant *negative* association with COVID-19 mortality at ages 25+. This relationship was concentrated at ages 65+, where a one-unit increase in COVID-19 mortality was found to be associated with a 0.037 unit decrease in cancer mortality. Thus, on average, individuals living in states with higher burdens of COVID-19 mortality also faced declining mortality from cancer.

We observe no association at the state level between COVID-19 mortality and changes in mortality from external causes [**Table 3**]. To investigate whether the aggregate of external causes is obscuring important relationships involving more detailed external causes of death, we conducted a supplementary analysis that examined mortality from drug overdose, homicide, suicide, transport accidents, and all other external causes of death presented in the S1 Appendix. As shown in **S2 Table in S1 Appendix**, none of the changes in mortality from external causes was significantly related to mortality from COVID-19.

**Fig 3:**
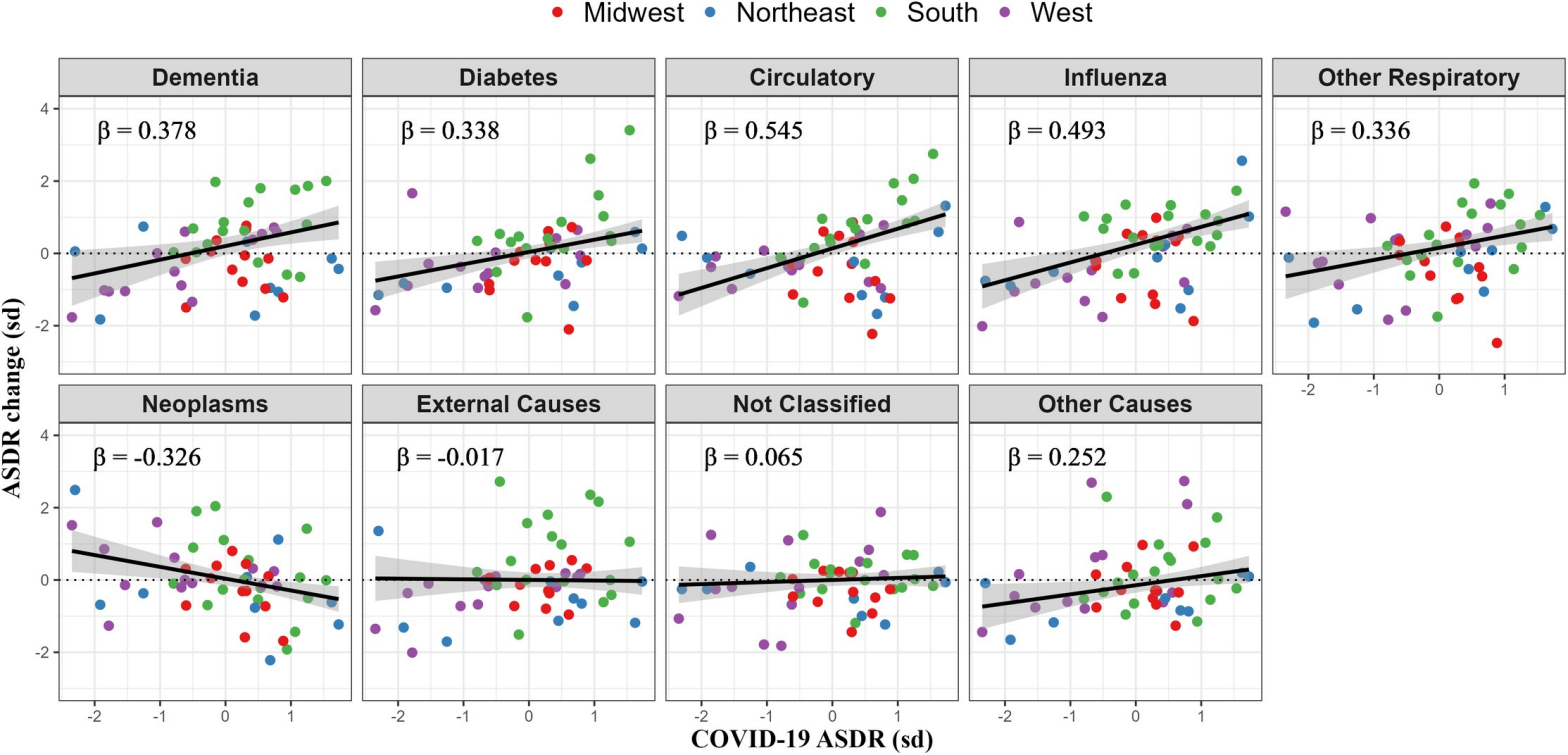
Standardized association between state-level COVID-19 mortality and change in cause-specific mortality, ages 25+. Note: ASDR = age-standardized death rates. Change is measured as the age-standardized death rate in first full year of the pandemic (March 2020 to February 2021) relative to the same period a year prior (March 2019 to February 2020), with coefficients standardized (mean = 0 and standard deviation = 1). Line reflects weighted linear regression where weights are the corresponding size of population in state i. Points above the dotted line indicates states with increases in cause-specific age ASDR, while below indicates decreases. *β* represents the change in ASDR for a particular cause of death in SD units associated with a 1 SD unit increase in COVID-19 ASDR.

The associations between COVID-19 mortality and mortality from other causes of death can also be expressed in standard deviation units, i.e., using standardized beta coefficients, which introduce comparable scales for examining the strength of relationships across causes of death. These coefficients are presented in **Table 4** and **Fig 3**. The ordering of causes of death in terms of the strength of associations is fairly similar to that in **Table 3**, with circulatory disease showing the highest coefficient in both cases. However, influenza/pneumonia, a cause of death with relatively low mortality, emerges as having the second highest beta coefficient when measured in standard deviation units. In fact, among the working-age population (25–64), the beta coefficient on influenza/pneumonia appears to have an even a stronger association with COVID-19 mortality than circulatory disease, a cause of death with relatively high mortality. Thus, once a comparable scaling of their death rates is imposed, death rates from the two infectious diseases are seen to be closely related across states.

**Table 4:** Regression of state-level change in cause-specific mortality on COVID-19 mortality, standardized coefficients.

	Ages 25+			Working Ages			Older Ages		
				(25–64)			(65+)		
	α	β	p-value	α	β	p-value	α	β	p-value
Dementia	0.20	0.38	0.008	-0.09	0.14	0.13	0.24	0.31	0.03
Diabetes	0.04	0.34	<0.001	-0.10	0.27	0.001	0.14	0.27	0.02
Circulatory Disease	0.14	0.55	<0.001	-0.08	0.29	0.009	0.25	0.45	<0.001
Influenza & Pneumonia	0.24	0.49	<0.001	0.03	0.38	0.002	0.28	0.40	0.001
Other Respiratory	0.15	0.34	0.005	0.20	0.25	0.02	0.14	0.22	0.06
Malignant Neoplasms	0.04	-0.33	0.003	0.02	0.07	0.45	0.01	-0.46	<0.001
Signs Not Classified	0.01	0.07	0.52	0.11	0.21	0.10	-0.05	0.04	0.68
External Causes	0.00	-0.02	0.88	0.00	0.01	0.91	-0.04	0.03	0.75
Other	-0.15	0.25	0.03	-0.24	0.10	0.26	0.01	0.26	0.03

Note: Table presents OLS regression of state-level absolute change in cause-specific age-standardized death rates (per 100,000) in the first full year of the pandemic (March 2020 to February 2021) relative to the same period a year prior (March 2019 to February 2020) on the COVID-19 age-standardized death rate in the first year of the pandemic. Regressions were weighted by the state population in each age group. Coefficients are standardized (mean = 0 and standard deviation = 1).

### Regional trends

Further, the state-level analyses also suggestively reveal regional differences between actual and predicted non-COVID-19 mortality, informing our understanding of the spatial patterning of excess mortality across the United States. The state-level relationship illustrated in the results highlights clear regional patterns in the states that either experienced unexpectedly high or low death rates from COVID-19 mortality relative to their change in non-COVID-19 death rates, with a clear divergence between the states located in the South and Northeast. As evident from **Fig 2**, Southern states appear to dominate the positive residuals, with all states in the region with one exception lying above the fitted regression line of non-COVID-19 mortality on COVID-19 mortality. This analysis suggests that Southern states generally had more non-COVID-19 mortality than would be predicted based on their COVID-19 mortality during the first year of the pandemic. For example, Mississippi (MS), which faced some of the highest rates of COVID-19 mortality in the country, experienced increases in non-COVID-19 mortality approximately double the level predicted by our analysis [**Fig 2**].

Conversely, all but one of the states located in the Northeast lie below the regression line, suggesting these states saw smaller increases in non-COVID-19 mortality than anticipated based on the levels of COVID-19 mortality experienced during the first year of the pandemic. Massachusetts (MA) serves as a clear example of this dynamic. Despite facing COVID-19 death rates of nearly 120 per 100,000 in the first year of the pandemic, which would correspond in our analysis to an increase in non-COVID-19 mortality of over 25 per 100,000, the state actually experienced a decrease in non-COVID-19 mortality during the time period. Additionally, this regional patterning appears to be largely consistent across the non-COVID-19 causes of death examined in the study, but particularly so for dementia, circulatory diseases, and external causes [**Fig 2**].

## Discussion

This study estimates that changes in mortality from causes of death other than COVID-19 represent 19.6% of the total change in mortality between March 2019-February 2020 and March 2020-February 2021 **[Table 1]**. This estimate of the effects of the COVID-19 pandemic on other causes of death is in line with estimates by the Center for Disease Control that suggest that the contribution of non-COVID-19 deaths range from 12% to 25% of the total change in mortality. The causes of death that contribute the most to the increase in all-cause mortality associated with mortality from COVID-19 are circulatory diseases and dementia. Other causes of death making large contributions are other respiratory diseases (including chronic obstructive pulmonary disease), influenza/pneumonia, and diabetes. Circulatory disease, dementia, and diabetes were also the three causes of death showing the largest mortality increase in the nation between 2019 and 2020, apart from external causes and COVID-19 itself [35]. Below we consider several possible explanations for a positive association between COVID-19 mortality and mortality attributed to the natural causes of death.

### Changes in health care utilization

First, positive associations may relate to the fact that states with high COVID-19 death rates may have had larger declines in health care utilization than states with low COVID-19 death rates due to interruption and delays in the provision of health care services and greater hospital avoidance [33–35]. Chronic conditions for which management requires frequent medical monitoring, such as diabetes and circulatory disease, are likely to be most affected by these reductions [36]. These interruptions may have been exacerbated by increased housing and food insecurity brought on by the pandemic, an effect that may be particularly salient among those living with chronic illnesses or who face acute health emergencies and cannot afford medicines or medical supplies [37–39]. Further, some similar infectious diseases that spread by respiratory routes may be highly correlated across states because they respond to the same set of environmental influences, including shelter-in-place and masking policies. For example, influenza/pneumonia shows the second closest association with COVID-19 mortality when associations are measured in standard deviation units.

### Cause-of-death coding practices

Additionally, diagnostic and coding practices may have resulted in COVID-19 deaths being inappropriately assigned to another underlying cause. CDC guidelines suggest that, if COVID-19 were listed on a death certificate, it should be classified as the underlying cause of death, with pre-existing conditions listed as contributing causes of death [40]. Instead, during the first year of the pandemic, 13% of death certificates that included COVID-19 had it placed as a contributing cause of death [41]. If the proportion of deaths that should have been assigned to COVID-19 but are instead assigned to another underlying cause is roughly constant from state to state, it would create a positive association between COVID-19 mortality and mortality from that other cause. Such a pattern could be obscured or even reversed if there were sufficient interstate variability in diagnostic and coding practices.

Coding confusions are particularly likely when there are synergies between COVID-19 infection and another disease. By synergies, we refer to physiological relations between COVID-19 and a medical condition such that the mortality risk from joint exposure to the two conditions exceeds the sum of risks from the individual exposures. Synergies may also be present by virtue of damage that COVID-19 infection does to various organ systems among people with no prior disease [42–44].

Some prior studies help to cast light on the presence or absence of synergies for certain causes of death. Using data on English cohorts, one such study compared the odds ratio of death rates for people with a particular condition in 2020, when COVID-19 was present, to ratios in 2015–2019, when it was not [45]. Conditions that raised the relative odds of death during the pandemic were dementia, diabetes, hypertension, stroke, and coronary heart disease (insignificantly), while cancer and chronic obstructive pulmonary disease (COPD) had lower odds ratios in 2020 than in 2019. Similarly, Tarazi et al. (2021) use data from Medicare beneficiaries that permit the construction of rate ratios in the two periods, finding enrollees with dementia, diabetes, hypertension, cardiac disorders, and COPD were at greater relative risk in 2021 than in 2015–19 [46]. However, as in the English study, Medicare enrollees with cancer were at lower relative risk in 2020 than in the prior period (2015–19), possibly implying an absence of a cancer synergy with COVID-19. A handful of studies have begun to provide additional evidence of synergies between COVID-19 and particular causes of death, including coronary heart disease or cardiovascular disease [47, 48].

### Malignant neoplasms: An anomaly

Interestingly, we find that malignant neoplasms do not behave like other chronic diseases in their relation to COVID-19. States with higher death rates from COVID-19 instead had smaller increases, or larger declines, in mortality from cancer than states with lower mortality from COVID-19. Although somewhat surprising, this finding is not inconsistent with emerging evidence on this topic. Mortality from cancer at the national level has also proved anomalous, showing no change between 2019 and 2020 at ages 25+ while mortality from other major causes was rising [49]. These findings align with the studies mentioned above [45, 46], which found that individuals with cancer did not suffer from unusually high relative risks of death during 2020, the first year of the pandemic, compared to 2015–2019. Interestingly, this experience is also consistent with that of the 1918 influenza epidemic in the United States, when the outbreak was found to be correlated with higher mortality from respiratory and heart disease, but not with that from cancer [45].

However, an absence of synergy between cancer and COVID-19 would not explain why cancer mortality *fell* in the states with the highest burden of COVID-19 mortality. One hypothesized mechanism that would create this negative association between mortality from COVID-19 and other causes of death, such as cancer mortality, can be termed frailty selection [50]. It is plausible that frailer cancer patients would be more likely to die from COVID-19 than less frail patients when infected, resulting in a remaining cancer population that is somewhat less frail as a result of the pandemic and thus less likely to die from cancer. Although such a process of selection should have been operating with respect to the other diseases as well, its importance may have been obscured by the power of disease synergies, perhaps expressed through greater degrees of diagnostic and coding error. It has also been suggested that, in view of the seriousness of the condition, cancer patients may have been more likely to shield or be compliant with social distancing measures [49]. In states with higher COVID-19 transmission and more COVID-19 deaths, cancer patients may have sheltered-in-place at higher rates than cancer patients in places with less COVID-19 transmission.

Nonetheless, the relationship between cancer and COVID-19 during the pandemic remains surprisingly unclear. Emerging work has shown that heterogeneity in cancer types, as well as variation in cancer epidemiology and practice across countries, have vastly complicated our empirical understanding of this relationship [51, 52]. This study thus adds to the growing body of literature that calls for more research on this topic so that we may better understand the link between cancer and COVID-19.

### External causes of death

Another anomalous cause of death observed in our study is external causes. Research has consistently documented a sharp increase in death rates from external causes during 2020 [8, 12, 32, 53]. This work has pointed to pandemic-related disruptions and hardships as responsible for increases in stress, depression, and substance use, contributing to increased mortality from drug overdose and alcohol abuse [54, 55], as well as traffic accidents [56]. Our results, however, show no association between mortality from COVID-19 and that from external causes across states [**Table 2**].

It has been pointed out that mortality from drug overdose, a major component of external causes, was rising before the pandemic and that some of the increase in 2020 and 2021 from external causes may reflect a continuation of that trend [17, 19, 20, 54]. Our results suggestively support this interpretation: there is no observed “dose/response” relationship between COVID-19 mortality and mortality from external causes. Instead, there is a very large positive intercept in the external cause regression, the largest for any cause of death at both ages 25+ and 25–64 [**Table 2**]. This is particularly true for drug overdose deaths among working aged populations [**Table 3**]. The intercept is an indicator of what changes in mortality from external causes would have been for populations in a state where the death rate from COVID-19 were set at zero, i.e., where there was no COVID-19 mortality experienced during the first year of pandemic. The implication is that external cause mortality would have risen substantially over the period even in the absence of the COVID-19 pandemic.

However, the fact that external cause mortality appears unresponsive to the level of COVID-19 mortality in a state does not imply that there was no impact of the pandemic on external causes of death, only that the relationship between COVID-19 and external cause mortality did not have a prominent spatial component. There are likely indirect effects of COVID-19 that were “universal” across the country that would not be captured in the spatial relationships in our regressions. This may be particularly true for external cause mortality, where increases in certain causes of death–such as drug overdose and homicide–may be partly in response to the pandemic-related economic hardship and associated stress experienced on a national scale [57].

### Other explanatory factors

This discussion does not exhaust the possible reasons why states with higher levels of mortality from COVID-19 would have greater increases in mortality from other causes of death. Over the past several decades, a large body of research has emerged to examine the vast array of sociodemographic, behavioral, environmental, and institutional factors that drive differences in health and mortality across states in the United States [17, 19, 20, 58]. Within the context of the COVID-19 pandemic, many of the factors operating at a state level that raised or lowered death rates from COVID-19 may also be expected to have raised or lowered death rates from another cause.

These could include state-level variation in the extent of co-morbidities, such as obesity, diabetes, and heart disease [59]. For example, the well-documented “stroke belt’ of the Central South, where cardiovascular diseases are highly concentrated, may underlie the spatial patterns that emerge between circulatory diseases and COVID-19 mortality observed in the study [60, 61]. Additionally, prior work has pointed to other pandemic-related factors, such as a state’s efforts to control the pandemic through public health measures [59, 60] or the ability of its healthcare systems to react to the pandemic [62]. These factors, coupled with broader state-level variation in socioeconomic status, racial and ethnic composition, and population density are likely to collectively shape the unequal impact of the pandemic across states [63, 64]. Although conducting a full-scale multivariate analysis of state-specific circumstances that fashioned the mortality response to the pandemic is beyond the scope of this paper, examining the factors that drive the spatial patterns in mortality for major causes of death observed in this study is a promising avenue for future research.

### Limitations

We recognize several limitations in this analysis. First, the full impact of the COVID-19 pandemic on health and mortality may be experienced with a lag, while this analysis solely examines the relationships that emerged in the first full year of the COVID-19 pandemic. For example, delays in diagnoses driven by the pandemic’s disruption of health services may serve to impact mortality primarily in the long-term, which could be missed in the present study given the focus on the first full year of the pandemic [65, 66].

Second, interstate variation in diagnostic and coding practices may work to obscure the true relationship between mortality from COVID-19 and another cause of death. If an absence of diagnostic testing in some places caused certifiers to systematically list a co-morbid condition, rather than COVID-19, as the underlying cause, death rates from comorbid conditions would be inflated while those from COVID-19 would be deflated relative to places where certifiers had full information. Such variation in coding practices is capable of creating an inverse association between COVID-19 and another cause of death. For example, Massachusetts may represent a concrete instance of unusual coding practices. We document unexpectedly high death rates from COVID-19 in Massachusetts relative to the change in non-COVID-19 death rates (**Fig 1**). This result raises the possibility that deaths from COVID-19 are over-recorded in Massachusetts, a possibility first suggested by Ackley et al [10]. Subsequent developments confirm that Massachusetts was using criteria for a COVID-19 death that were much more liberal than those which were used in other states [67]. Similarly, the fact that we observe Southern states consistently above the regression line predicting non-COVID-19 mortality raises the possibility that non-COVID-19 mortality was systematically higher than recorded in the South, suggesting that many COVID-19 deaths went uncounted there. Despite these apparent regional disparities in coding, the fact that relations between COVID-19 and mortality from most other cause of death are positive is consistent with the idea that states’ classification practices are not dissimilar enough to create inverse relationships. More research is needed to fully understand how inconsistencies in diagnostic and coding practices across states may shape the observed associations between COVID-19 and other causes of death.

Additionally, since mortality data was extracted by age group, state of residence, and cause of death, there is likely some degree of data suppression for less common causes of death in less populous states. We mitigate this concern through the use of weighted regressions, where each state is weighted by the corresponding size of the state’s population, thus reducing the influence of smaller states where suppression is more likely to occur and better reflecting the population distribution of the United States.

Finally, it is possible that the relationship between mortality from COVID-19 and other causes of death changed over the course of the pandemic, particularly across the various peaks in COVID-19 mortality. This may be particularly true as testing became more widespread and coding practices more established. However, given the already granular extraction of mortality data by age group, state, and cause of death, the study focused on mortality changes experienced in the first full year of the pandemic. Future research should investigate the extent to which these findings hold across distinct COVID-19 mortality peaks experienced during the pandemic.

Despite these limitations, this study takes a novel approach to understanding the full impact of the COVID-19 pandemic on mortality in the United States and is among the first to examine spatial patterns in mortality for major causes of death during the pandemic. In theorizing the possible mechanisms that underlie the pandemic’s impact on mortality from non-COVID-19 causes, this study contributes to a more complete understanding of how the COVID-19 pandemic may have directly and indirectly shaped the landscape of mortality in the United States.

## Supporting information

S1 Appendix (DOCX)
