# Supplementary material for: Associations between mortality from COVID-19 and other causes: A state-level analysis
Source: PLoS One. 2023 Mar 6;18(3):e0281683. doi: 10.1371/journal.pone.0281683 (PMC9987806; doi:10.1371/journal.pone.0281683)
Supplement: S1 Appendix — (DOCX) [file pone.0281683.s001.docx]

**Supplementary Appendix**

**S1 Table. List of Included Causes of Death and ICD-10 Codes**

**S2 Table. Regression of state-level change in mortality for select external causes on COVID-19 mortality**

**S1 Table. List of Included Causes of Death and ICD-10 Codes**

| **Causes of Death, Primary Analysis** | **ICD-10 Codes** |
| --- | --- |
| All Cause Deaths | All Codes |
| Covid-19 | U07.1 |
| Non-Covid-19 | All (except U07.1) |
| Dementia | F01-F04, G30, G31 (except for G31.2) |
| Diabetes | E10-E14 |
| Circulatory Disease | I00-I99 |
| Influenza & Pneumonia | J09-J18 |
| Malignant Neoplasm | C00-C97 |
| Other Respiratory Diseases | J00-J06, J20-J98 |
| Signs Not Classified | R00-R99 |
| External Causes | V01-Y89 |

| **Causes of Death, Supplemental Analysis** | **ICD-10 Codes** |
| --- | --- |
| Drug Overdose | X40-X44, X85, Y10-Y14 |
| Homicide | X86-X99, Y00-Y09, Y87.1 |
| Suicide | X60-X84, Y87.0 |
| Transport Accidents | V01-V99, Y85 |

**S2 Table. Regression of state-level change in mortality for select external causes on COVID-19 mortality**

|  | **Ages 25+** | | |  | **Working Ages  (25-64)** | | |  | **Older Ages  (65+)** | | |
| --- | --- | --- | --- | --- | --- | --- | --- | --- | --- | --- | --- |
|  | **α** | **β** | **p-value** |  | **α** | **β** | **p-value** |  | **α** | **β** | **p-value** |
| Drug Overdose | 5.76 | -0.012 | 0.32 |  | 6.38 | -0.027 | 0.50 |  | 0.95 | -0.001 | 0.63 |
| Homicide | 0.52 | 0.004 | 0.14 |  | 0.91 | 0.010 | 0.22 |  | 0.25 | 0.000 | 0.39 |
| Suicide | -1.15 | 0.006 | 0.06 |  | -0.92 | 0.011 | 0.24 |  | -1.14 | 0.002 | 0.12 |
| Transport Accidents | 0.40 | 0.000 | 0.99 |  | 0.73 | 0.004 | 0.68 |  | -0.32 | -0.002 | 0.29 |
| Other External Causes | 0.93 | -0.001 | 0.80 |  | 0.58 | 0.003 | 0.71 |  | 0.61 | 0.002 | 0.67 |

^ p < 0.10. Table presents OLS regression of state-level absolute change in cause-specific age-standardized death rates (per 100,000) in the first full year of the pandemic (March 2020 to February 2021) relative to the same period a year prior (March 2019 to February 2020) on the COVID-19 age-standardized death rate in the first year of the pandemic. Regressions were weighted by the state population in each age group.
